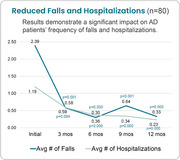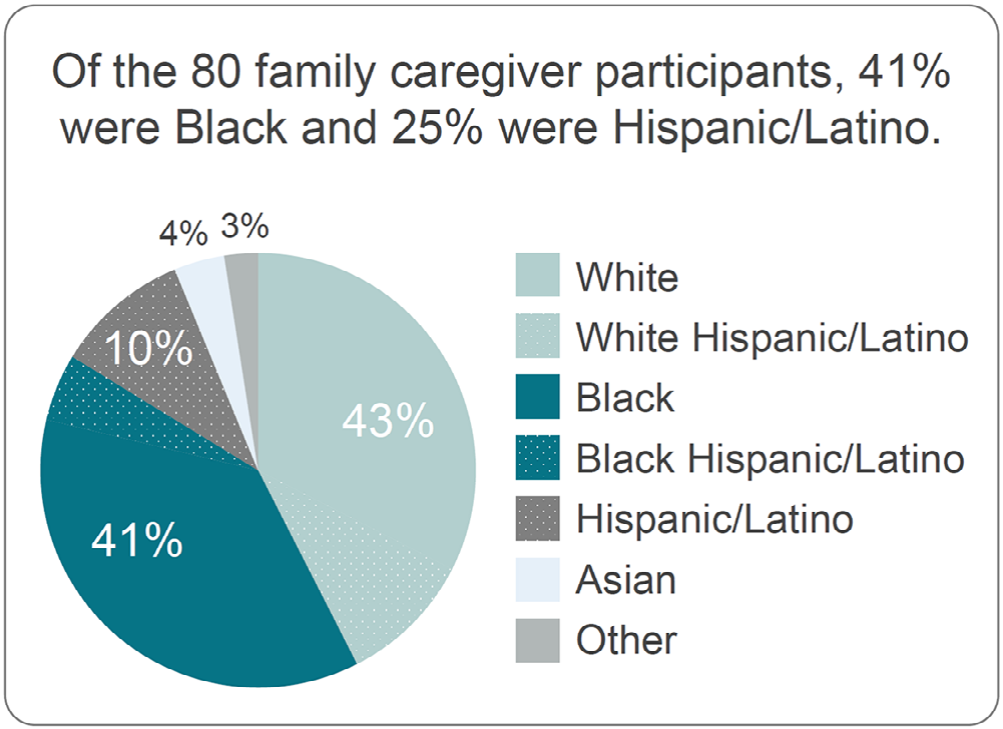# Virtual Support for Family Caregivers Reduces Hospitalizations and Falls in People with Alzheimer’s Disease: Results of a 12‐month Pilot Program

**DOI:** 10.1002/alz70861_108710

**Published:** 2025-12-23

**Authors:** Rosemary D. Laird, Cari Eyre, Jason Abrevaya

**Affiliations:** ^1^ NAN Navigator Inc., Orlando, FL USA; ^2^ Department of Elder Affairs, Tallahassee, FL USA; ^3^ University of Texas‐Austin, Austin, TX USA

## Abstract

**Background:**

The Florida Department of Elder Affairs and Navigating Aging Needs, LLC (NAN) have formed a public‐private partnership to provide virtual support to family caregivers of people with Alzheimer’s disease and related disorders (ADRD) living at home. The program targets a diverse population of patients with high‐level daily care needs, increased risk for future decline, and the need for costly Medicaid‐supported care.

**Objectives:**

To reduce the risk of falls and hospitalizations for people living with ADRD.

**Methods:**

Family caregivers of an individual with ADRD who qualifies for state assistance were offered a 12‐month support program. Support included regular meetings (Zoom or phone) with a personal licensed social worker. The initial assessment included 80 questions on the ADRD patient’s medical, emotional, social, and legal/financial well‐being and the validated battery of 12 questions comprising the short form Zarit Burden Interview scale (ZBI‐12). Following the assessment, the social worker provided the family caregiver with a personal plan that identified areas of risk that “need attention” or “may need attention,” along with resources to address identified needs. For the 12‐month pilot, the social worker met with the family caregiver regularly to help resolve areas identified as high risk and discuss other relevant issues as they arose. Every three months, the social worker conducted a reassessment with the family caregiver.

**Results:**

Data are available for 80 family caregivers receiving virtual support through the pilot for twelve months. Results demonstrate a significant impact on ADRD patients’ frequency of falls and hospitalizations among a racially diverse cohort. ADRD patient falls were reduced by 86% and hospitalizations by 81%. Results also demonstrate significantly reduced ZBI‐12 scores for family caregivers.

**Conclusion:**

Providing virtual support to family caregivers of people with Alzheimer’s disease reduces falls and hospitalizations for care recipients with Alzheimer’s disease and reduces caregivers’ stress. The Florida Department of Elder Affairs and NAN continue to evaluate the program’s impact on family caregivers, people with ADRD, and the cost of supporting this complex population.